# Advances in Transcatheter Mitral Valve Replacement (TMVR) in Patients with Mitral Annular Calcification: A Case Report of Acute Hemolytic Anemia and Review of Contemporary Approaches

**DOI:** 10.3390/jcm14134660

**Published:** 2025-07-01

**Authors:** Natalia Fongrat, Umang Makhijani, Nivetha Vajayakumar, Andrew Mangano, Micaela Iantorno

**Affiliations:** 1 Internal Medicine, Mary Washington Healthcare, Fredericksburg, VA 22401, USA; umang.makhijani@mwhc.com (U.M.); nivetha.vijayakumar@mwhc.com (N.V.); andrew.mangano@mwhc.com (A.M.); 2 Oracle Heart & Vascular, Fredericksburg, VA 22401, USA; micaela.iantorno@gmail.com

**Keywords:** MAC, TMVR, hemolytic anemia, LVOT obstruction, advances in transcatheter mitral valve replacement

## Abstract

Mitral valve disease, particularly in the context of extensive mitral annular calcification (MAC), poses significant challenges for traditional surgical management. Transcatheter mitral valve replacement (TMVR) has emerged as a promising alternative for high-risk and inoperable patients, driven by rapid advancements in valve technology, imaging techniques, and procedural strategies. Nevertheless, complications such as paravalvular leak (PVL), left ventricular outflow tract (LVOT) obstruction, and hemolysis remain obstacles to optimal outcomes, particularly in patients with complex annular anatomy. We present the case of an 89-year-old female with severe mitral stenosis and MAC who developed acute hemolytic anemia following experimental TMVR using the Edwards SAPIEN S3 valve. This case serves as a platform to explore recent advances in TMVR, including novel device platforms, enhanced imaging modalities for pre-procedural planning, innovative deployment strategies, and emerging adjunctive techniques aimed at reducing complications. Through this case, we underscore persistent challenges and emphasize the importance of meticulous patient selection and vigilant follow-up. Despite substantial progress, TMVR in the setting of MAC remains high-risk, demanding continued innovation in valve design, refined patient stratification, and improved peri-procedural management to enhance outcomes and mitigate risks such as hemolysis.

## 1. Introduction

Mitral annular calcification (MAC) is a chronic degenerative process characterized by calcium deposition in the fibrous ring of the mitral valve. While often asymptomatic, severe MAC can lead to mitral valve dysfunction, including stenosis and regurgitation, and is associated with increased morbidity and mortality [1]. Surgical mitral valve replacement in patients with severe MAC poses significant challenges due to the risk of annular rupture, atrioventricular disruption, and difficulty in securing the prosthesis, often resulting in high perioperative mortality rates [2,3].

Transcatheter mitral valve replacement (TMVR) has emerged as a less invasive alternative for high-risk patients with severe MAC who are poor candidates for conventional surgery. TMVR involves implanting a prosthetic valve via catheter-based techniques, avoiding the need for open-heart surgery. However, TMVR in the setting of MAC (ViMAC) presents unique challenges, including the risk of left ventricular outflow tract (LVOT) obstruction, paravalvular leak, valve embolization, hemolytic anemia, and difficulty in achieving secure anchoring due to the heavily calcified and irregular annulus [4].

Recent technological advances have propelled TMVR into the forefront of structural heart interventions, particularly for patients deemed inoperable due to prohibitive surgical risk [1,2]. These innovations include enhanced valve platforms, improved delivery systems, and refined imaging modalities that allow for precise anatomical assessment and procedural planning [3,5]. Over the past three years, several studies have highlighted significant progress in TMVR technologies, including the introduction of self-expanding and mechanically anchored devices, as well as adjunctive techniques aimed at reducing complications such as paravalvular leak (PVL) left ventricular outflow tract (LVOT) obstruction, and hemolysis [6,7,8].

This manuscript seeks to provide a comprehensive overview of the current landscape of TMVR in the setting of MAC, illustrate an acute case of hemolytic anemia following TMVR, and examine recent advancements in device design, procedural techniques, and imaging technologies that are redefining treatment approaches for this high-risk patient population.

## 2. Case Presentation

An 89-year-old woman with a history of hypertension, hyperlipidemia, and severe aortic stenosis initially presented with progressive exertional dyspnea. She underwent a transcatheter aortic valve replacement (TAVR) with a 29 mm Medtronic CoreValve. Although the procedure was technically successful, she experienced only partial symptomatic improvement. Subsequent evaluation revealed severe mitral stenosis with extensive mitral annular calcification (MAC), and she was deemed a poor surgical candidate. Three years later, she underwent an experimental transcatheter mitral valve replacement (TMVR) using a 29 mm SAPIEN Ultra RESILIA with LAMPOON.

The TMVR procedure was technically successful. However, within several days, the patient developed symptoms concerning for a new complication, including worsening fatigue and dyspnea. On physical examination, she had pale conjunctiva, scleral icterus, and mild jaundice. Pulmonary auscultation revealed bibasilar crackles, and trace bilateral lower extremity edema was noted, consistent with mild volume overload. A laboratory evaluation showed a decline in hemoglobin from 13 g/dL to 10.5 g/dL, elevated lactate dehydrogenase (LDH), low haptoglobin, elevated indirect bilirubin, and an increased reticulocyte count suggestive of bone marrow compensation. A peripheral blood smear revealed schistocytes, and Coombs testing was negative. These findings confirmed intravascular hemolysis. Renal function and coagulation studies were within normal limits. Stool guaiac testing was negative. Transthoracic echocardiography demonstrated preserved biventricular systolic function with a mean gradient of 6 mmHg across the mitral prosthesis—within the expected range but still concerning for shear-related red blood cell destruction. A diagnosis of prosthesis-related hemolytic anemia was made. She was managed conservatively with transfusions and fluid optimization. Her condition stabilized, and she was discharged to a rehabilitation facility.

Two weeks later, she re-presented with recurrent dyspnea, fatigue, and melena with diarrhea. Her hemoglobin had further declined to 8.7 g/dL. While gastrointestinal blood loss was considered, esophagogastroduodenoscopy (EGD) was unremarkable, and an outpatient colonoscopy was arranged. Repeat echocardiography again demonstrated preserved cardiac function and a stable transvalvular gradient. Hematology and cardiology teams were reconsulted. Other causes of anemia were ruled out, and prosthesis-related hemolysis remained the most likely etiology.

Given her advanced age, frailty, and multiple comorbidities, she was not considered a candidate for valve reintervention. Despite medical management, including conservative measures to reduce hemolysis, the patient’s anemia and overall functional status continued to decline. After multidisciplinary discussion and shared decision-making with the patient and her family, she opted to transition to palliative care focused on comfort and quality of life.

This case highlights the importance of anticipating and mitigating mechanical complications such as hemolytic anemia following TMVR, particularly in patients with severe mitral annular calcification. Prevention strategies include meticulous preprocedural imaging to guide valve sizing and positioning, avoidance of oversizing or malalignment that can increase shear stress, and thorough intraoperative assessment for paravalvular leaks or elevated transvalvular gradients. Emerging techniques, including the use of TMVR devices specifically designed for MAC anatomy and advanced procedural planning with 3D echocardiography or cardiac CT, may reduce the risk. Early postprocedural monitoring with hemolysis labs and echocardiography is also essential for timely diagnosis and intervention.

## 3. Advances in Transcatheter Mitral Valve Replacement: A Contemporary Review

### 3.1. Advancements in Device Design

TMVR has evolved considerably in response to the growing need for less invasive mitral interventions, particularly among patients with severe mitral annular calcification (MAC) or prior cardiac surgeries. The initial TMVR approaches leveraged balloon-expandable aortic valves, such as the Edwards SAPIEN series, repurposed for implantation in the mitral position. While these devices demonstrated technical feasibility, they encountered substantial procedural challenges, primarily related to the anatomical complexities of the mitral annulus. Unlike the relatively circular and rigid structure of the aortic annulus, the mitral annulus is more elliptical and often heavily calcified in patients with MAC, complicating secure anchoring and adequate sealing. This mismatch frequently led to paravalvular leak (PVL), valve embolization, and left ventricular outflow tract (LVOT) obstruction—complications that significantly impact clinical outcomes and procedural success rates [1,2,3].

To address these limitations, the field has witnessed the development of purpose-built TMVR devices specifically designed to accommodate the anatomical variability and calcification present in the mitral position. Among the most notable advancements is the Tendyne Mitral Valve System (Abbott), which utilizes a self-expanding porcine pericardial valve secured via an apical tether. This tethering mechanism not only facilitates stable positioning within the annulus but also allows for retrieval and repositioning if required—an important feature given the dynamic anatomical environment of the mitral valve. Additionally, the Tendyne system’s design reduces the risk of PVL by conforming more effectively to the irregular calcified annulus [5].

Another significant innovation is the Intrepid TMVR System (Medtronic), which incorporates a dual stent frame architecture. This design includes an inner valve for flow regulation and an outer fixation ring that provides enhanced anchoring and stability. The dual stent configuration allows the device to adapt more readily to varying annular geometries, thus minimizing the risks of PVL and embolization. Its self-expanding nature also facilitates secure engagement with the mitral annulus, even in cases of severe MAC, while mitigating LVOT obstruction through optimized flow dynamics [6,7,8,9,10].

In addition to these commercially available solutions, several investigational TMVR devices are currently undergoing clinical evaluation. The Cardiovalve is a promising example, designed with both innovative anchoring mechanisms and enhanced hemodynamic properties. The Cardiovalve TMVR system is an investigational device specifically designed for patients with severe mitral annular calcification (MAC). Its transfemoral-transseptal approach and dual-frame structure aim to enhance anchoring stability and minimize LVOT obstruction. Early feasibility studies have shown promising technical success and favorable hemodynamic outcomes in high-risk MAC populations. In a reported case, a 72-year-old male with recurrent heart failure due to severe mitral regurgitation and MAC underwent successful transfemoral Cardiovalve implantation. The procedure resulted in immediate hemodynamic improvement and symptom relief without complications such as left ventricular outflow tract (LVOT) obstruction and paravalvular leak [11]. A comparative summary of key features and design distinctions among these devices is presented in Table 1.

The AltaValve System, featuring an atrial fixation design, was assessed in a study involving six patients with severe MR and moderate to severe MAC. The device demonstrated technical success in all cases, with most patients showing significant improvement in MR severity and functional status at 30 days post-implantation. Notably, the atrial fixation approach aims to minimize the risk of LVOT obstruction [12].

Furthermore, a multicenter registry study examined the transatrial valve-in-MAC (ViMAC) approach in 126 patients. The procedure achieved a 94.4% technical success rate, with low incidences of paravalvular leak. However, the study reported 30-day and one-year all-cause mortality rates of 12.7% and 26.2%, respectively. These findings suggest that while the transatrial ViMAC approach is feasible, patient selection remains critical to optimize outcomes [13].

These advancements underscore the significant progress in TMVR technology, shifting from repurposed aortic valves to purpose-built solutions that address the unique anatomical and hemodynamic challenges of the mitral valve. This evolution not only enhances procedural safety but also extends the therapeutic options available for high-risk patients previously deemed unsuitable for surgical intervention.

### 3.2. Pre-Procedural Imaging and Planning

Recent advancements in pre-procedural imaging and planning for TMVR in patients with mitral annular calcification MAC have significantly enhanced procedural success and patient outcomes. Multidetector computed tomography (MDCT) is now the preferred imaging modality for evaluating the mitral valve apparatus. Its high-resolution capabilities allow for detailed assessment of MAC’s extent, precise measurement of annular dimensions, and accurate prediction of the post-implantation neo–LVOT area—an essential factor in identifying and mitigating the risk of LVOT obstruction [9,14]. MDCT’s ability to provide comprehensive anatomical visualization aids in planning optimal access routes, assessing landing zones, and anticipating potential challenges during device deployment.

In addition to static imaging, virtual modeling and simulation technologies have become instrumental in the pre-procedural planning of TMVR. These advanced tools enable heart teams to virtually “implant” the device, simulate its interaction with the heavily calcified annulus, and predict procedural outcomes. Such simulation-based approaches facilitate precise device sizing and optimal positioning, reducing the risk of PVL, malposition, and valve embolization. This forward-looking strategy allows interventionalists to refine procedural steps, anticipate anatomical complications, and optimize valve selection for each patient’s unique anatomy. Studies have demonstrated that three-dimensional (3D) prototyping and computational modeling can effectively simulate potential complications, such as LVOT obstruction, and assist in selecting appropriate device sizes and implantation strategies [15].

Intra-procedural imaging has also evolved, with three-dimensional transesophageal echocardiography (TEE) playing a pivotal role in real-time guidance. TEE enhances visualization during device alignment and deployment, allowing for the immediate detection of complications such as PVL, malposition, and pericardial effusion. Furthermore, the integration of fluoroscopy and echocardiography through fusion imaging techniques has revolutionized procedural accuracy. This hybrid imaging approach enables the synchronized visualization of both anatomical landmarks and device positioning, resulting in improved precision and reduced procedural risks [11]. Collectively, these advancements in imaging and planning have not only improved safety and efficacy in TMVR for MAC patients but also expanded the procedural possibilities for this high-risk group [15,16].

### 3.3. Innovations in Procedural Techniques

Innovations in procedural techniques have helped overcome the specific technical challenges associated with mitral annular calcification (MAC). In certain cases, pre-procedural interventions such as balloon valvuloplasty or intravascular lithotripsy have been employed to modify the heavily calcified and rigid annulus, thereby facilitating smoother valve delivery and reducing the risk of malposition [17]. Contemporary transcatheter mitral valve replacement (TMVR) devices are equipped with design enhancements such as extended skirts and sealing rings, which help conform to the irregular annular surface and significantly reduce the incidence and severity of paravalvular leak (PVL) [6,7].

A notable procedural innovation is the LAMPOON (Laceration of the Anterior Mitral leaflet to Prevent LVOT ObstructioN) technique, which was developed to address the potentially fatal complication of left ventricular outflow tract (LVOT) obstruction. This complication arises when the anterior mitral leaflet is displaced toward the LVOT during valve deployment, leading to dynamic obstruction. In the LAMPOON procedure, the anterior mitral leaflet is intentionally split along the midline using a transcatheter electrosurgical technique. Typically performed via a femoral venous approach, the procedure involves creating a guidewire rail between the femoral vein and artery, through which an electrified wire is positioned and energized to lacerate the leaflet under fluoroscopic and transesophageal echocardiographic (TEE) guidance. The controlled midline laceration enables the two halves of the anterior leaflet to splay open during valve expansion, thereby minimizing the risk of LVOT obstruction and allowing safe valve deployment in patients with high-risk anatomy [18,19].

Additionally, TMVR has been successfully adapted for valve-in-valve and valve-in-ring procedures in patients with degenerated surgical bioprostheses or annuloplasty rings, providing a less invasive alternative to surgical reoperation. These approaches utilize devices and sizing strategies specifically designed to accommodate the geometries of pre-existing valve structures, further broadening the scope of TMVR in complex mitral valve disease [20].

### 3.4. Selecting Appropriate Candidates for TMVR

The process of selecting appropriate candidates for TMVR is inherently multidisciplinary, requiring close collaboration among interventional cardiologists, cardiothoracic surgeons, imaging specialists, and anesthesiologists. Determining eligibility involves detailed evaluation of anatomical parameters—such as the extent and location of MAC, annular shape, and the projected neo–LVOT area, with values exceeding 1.7 cm^2^ generally considered favorable—as well as clinical considerations including ventricular function, comorbidity burden, frailty status, and anticipated life expectancy. The use of validated risk assessment tools and data from international TMVR registries has further enhanced personalized decision-making and contributed to improved procedural success [10,17].

### 3.5. Clinical Outcomes for TMVR

Although TMVR has seen notable advancements, particularly in patients with MAC, it continues to carry a higher risk of complications compared to other structural heart procedures. PVL remains the most common technical issue; however, improvements in valve design, implantation techniques, and the use of adjunctive measures such as percutaneous closure with plugs or coils have helped reduce its clinical burden [5,6]. Hemolytic anemia, often caused by PVL or turbulent, high-velocity regurgitant jets, can vary in severity from mild to life-threatening. Prompt diagnosis, leak correction, and supportive care are essential, though severe or unresponsive cases may require repeat intervention or surgical valve removal [5,6,16]. The incidence of LVOT obstruction—a previously significant contributor to mortality—has decreased due to thorough pre-procedural assessment and the implementation of strategies like the LAMPOON technique [18,19]. Thanks to enhanced imaging, improved device technologies, and growing procedural expertise, complications such as valve embolization and malposition have become increasingly uncommon.

Clinical outcomes for TMVR have steadily improved, with registry data and multi-center studies demonstrating that, in well-selected patients, TMVR can provide durable symptomatic relief and acceptable survival rates. Data from the MITRAL trial and other registries confirm that while technical and procedural success rates are rising, long-term durability, valve thrombosis, and structural degeneration remain concerns necessitating ongoing surveillance [3,19]. Device-specific registries and post-market surveillance efforts will be key as TMVR expands to broader patient populations.

The future of TMVR is set to undergo significant advancements. Continued progress in device technology—including systems that are fully repositionable and retrievable, enhanced sealing components, and customizable valve designs—is expected to improve procedural success and broaden the scope of treatable patients. The application of artificial intelligence and machine learning in pre-procedural imaging and patient risk stratification offers the potential to enhance safety, streamline decision-making, and better predict long-term outcomes [21,22]. Additionally, data from ongoing randomized clinical trials and the implementation of standardized post-procedural monitoring strategies will play a crucial role in refining patient care, reducing adverse events, and maximizing the therapeutic value of TMVR. As evidence accumulates and procedural experience deepens, TMVR is anticipated to become a more reliable and widely accepted treatment option for individuals with complex mitral pathology who are not candidates for traditional surgical repair or replacement.

Recent studies have provided valuable insights into the application of TMVR in MAC. The CHOICE-MI Registry, a significant multicenter study, evaluated 279 patients undergoing TMVR, stratifying them based on the severity of MAC. The study found that while technical success and mitral regurgitation (MR) resolution rates were similar across groups, patients with moderate to severe MAC experienced higher rates of postprocedural complications, such as bleeding and renal failure. At the two-year follow-up, survival rates and functional improvements were comparable between patients with and without significant MAC [23].

### 3.6. Limitations and Future Directions

TMVR has emerged as a promising intervention for patients with MAC. However, several limitations persist that impact procedural success and patient outcomes.

Device selection remains a significant challenge due to the heterogeneous and heavily calcified anatomy associated with MAC. This variability can complicate valve anchoring and increase the risk of PVL. LVOT obstruction is another serious complication, especially in patients with narrow neo-LVOT measurements post-implantation. The displacement of the anterior mitral leaflet during TMVR can exacerbate this risk [24].

The complexity of TMVR procedures necessitates meticulous pre-procedural imaging and real-time intraprocedural guidance, requiring advanced expertise and coordinated heart team collaboration. Despite technological advancements, the long-term durability of transcatheter mitral valves remains under investigation. Unlike surgical valves, the longevity of TMVR platforms has not been extensively studied, making long-term outcomes less predictable [25].

Hemolysis, as observed in the presented case, continues to be a concerning complication, often driven by high-velocity jets or PVL. Strategies to mitigate hemolysis, including enhanced valve design and adjunctive sealing mechanisms, are areas of active research.

Looking forward, future research directions should prioritize device innovation, including the development of fully repositionable and retrievable valve platforms, along with enhanced sealing mechanisms to minimize PVL and improve anchoring in calcified anatomies. Imaging and procedural planning should be enhanced through advanced imaging modalities, including fusion imaging and 3D virtual modeling, to optimize procedural planning and reduce complications.

Risk stratification and patient selection could benefit from the implementation of machine learning and artificial intelligence to refine patient selection criteria, predict procedural risk, and tailor device choice to individual anatomical features. Long-term surveillance is crucial, necessitating the establishment of robust registries and long-term follow-up studies to monitor valve performance, durability, and clinical outcomes.

Expanding the application of adjunctive techniques like LAMPOON (Laceration of the Anterior Mitral Leaflet to Prevent Outflow Obstruction) and intravascular lithotripsy to reduce anatomical barriers and improve procedural safety is another important focus. Ongoing clinical trials and registry data will be crucial in addressing these gaps, providing evidence-based guidelines to optimize TMVR outcomes for high-risk patients with MAC. Collaboration across clinical and engineering disciplines remains essential for overcoming current challenges and achieving the broader adoption of this transformative technology.

## 4. Conclusions

TMVR marks a transformative development in the treatment of high-risk mitral valve disease, providing a viable option for patients who were once deemed ineligible for intervention. Progress in device technology, advanced imaging, and procedural techniques has greatly enhanced the safety and effectiveness of TMVR, especially in challenging scenarios such as ViMAC. However, achieving the best possible outcomes still depends on meticulous patient selection, tailored procedural strategies, and thorough post-procedural monitoring. Ongoing research and technological innovation remain essential to addressing persistent challenges and reducing serious complications such as hemolytic anemia.

## Figures and Tables

**Table 1:** Comparison of TMVR Devices: Tendyne vs. Intrepid vs. Cardiovalve.

Feature	Tendyne	Intrepid	Cardiovalve
Developer	Abbott (Abbott Park, IL, USA)	Medtronic (Galway, Ireland)	Cardiovalve Ltd./Edwards Lifesciences (Or Yehuda, Central District, Israel)
Valve Type	Trileaflet porcine pericardial	Trileaflet bovine pericardial	Trileaflet bovine pericardial
Frame Design	Self-expanding nitinol, dual-frame	Self-expanding dual stent (inner + outer)	Multi-arm grasping frame
Delivery Approach	Transapical	Transapical (initially; transfemoral under study)	Transfemoral-transseptal
Fixation Mechanism	Apical tether anchoring to LV apex	Radial and axial fixation within mitral annulus	Grasping arms engage native leaflets
Annulus Sizing Range	−30–43 mm	−27–43 mm	−36–50 mm
Repositionability	Fully repositionable and retrievable	Repositionable before final release	Repositionable during deployment
LVOT obstruction avoidance	Moderate - depends on positioning	Designed to minimize LVOT obstruction	Low-profile valve to reduce LVOT risk
Clinical Trials	SUMMIT trial (ongoing)	APOLLO trial (ongoing)	AHEAD trial (ongoing)
CE Mark/FDA Status	CE Marked (EU), IDE ongoing (US)	Investigational (US and EU)	Investigational (early feasibility)
Special Notes	First TMVR device implanted in humans	Integrated delivery system, broad annular coverage	Designed for transfemoral-only access
